# Sustained serological and complete responses in HBeAg-positive patients treated with Peginterferon alfa-2b: a 6-year long-term follow-up of a multicenter, randomized, controlled trial in China

**DOI:** 10.1186/s12876-019-0981-5

**Published:** 2019-05-02

**Authors:** Jian Sun, Huiguo Ding, Guofeng Chen, Guiqiang Wang, Lai Wei, Jiming Zhang, Qing Xie, Mobin Wan, Hong Tang, Shijun Chen, Zhiliang Gao, Yuming Wang, Dazhi Zhang, Wenxiang Huang, Jifang Sheng, Qin Ning, Dongliang Yang, Jian Lu, Chen Pan, Yuxiu Yang, Jue Wang, Chuanzhen Sun, Qixin Wang, Jinlin Hou

**Affiliations:** 1 State Key Laboratory of Organ Failure Research, Guangdong Provincial Key Laboratory of Viral Hepatitis Research, Department of Infectious Diseases, Nanfang Hospital, Southern Medical University, Guangzhou, China; 2 Beijing You’an Hospital, Capital Medical University, No. 8, You An Men Wai Street, Fengtai District, Beijing, 100069 China; 3 Beijing 302 Hospital, No. 100, Xi Si Huan Zhong Lu, Beijing, 100039 China; 4 Peking University First Hospital, No. 8, Xicheng Xishiku Street, Beijing, 100034 China; 5 Peking University People’s Hospital, No. 11 Xizhimen South Street, Beijing, 100044 China; 6 Huashan Hospital, Fudan University, No. 12, Middle Wurumuqi Rd, Shanghai, 200040 China; 7 Shanghai Ruijin Hospital Affiliated Shanghai Jiao Tong University, No. 197, Ruijiner Rd, Shanghai, 200025 China; 8 Shanghai Changhai Hospital, No. 174, Changhai Rd, Shanghai, 200433 China; 9 Huaxi Hospital of Sichuan University, No. 37, Guo Xue Xiang, Chengdu, 610041 China; 10 Jinan Infectious Disease Hospital, No. 173, Jingshi Rd, Jinan, 250021 China; 11 The Third Affiliated Hospital of Sun Yat-sen University, No. 600, Tianhe Rd, Guangzhou, 510630 China; 12 Southwest Hospital Affiliated Third Military Medical University, No. 29, Gao Tan Yan Zhengjie, Shapingba, Chongqing, 400038 China; 13 Department of Infectious Disease, No. 2 Affiliated Hospital of Chongqing Medical University, No 74, Lingjiang Road, Yuzhong district, Chongqing, China; 14 Editing Department of Chinese Journal of Hepatology, No 74, Lingjiang Road, Yuzhong district, Chongqing, China; 15 No. 1 Hospital of Chongqing Medical University, No. 1, Youyi Rd, Chongqing, 400016 China; 16 The First Affiliated Hospital of Zhejiang University Medical College, No. 79, Qingchun Rd, Hangzhou, 310003 China; 17 Tongji Hospital Affiliated Tongji Medical College of Huazhong University of Science & Technology, No. 1095, Jiefang Avenue, Wuhan, 430030 China; 18 Department of Infectious Disease, Union Hospital, Tongji Medical College, Huazhong University of Science & Technology, 1277#, Jiefang Avenue, Wuhan, 430022 China; 19 Shenzhen Third People’s Hospital, Bulan Road 29, Longgong, Shenzhen, 518112 People’s Republic of China; 20 Fuzhou Infectious Disease Hospital, No. 312, Xihong Rd., Gulou District, Fuzhou, 350025 China; 21 Henan Provincial People’s Hospital, No. 7, Weiwu Rd, Zhengzhou, 450003 China; 22 MSD China, Building A, Headquarters Park, Phase 2, 1582 Gumei Road Xuhui District, Shanghai, 200233 China

**Keywords:** Chronic hepatitis B, Peg-interferon, Long-term follow-up, Sustained serological response, Sustained combined clinical response

## Abstract

**Background:**

Pegylated interferon (PEG-IFN) alfa-2b is recommended for chronic hepatitis B (CHB). We aimed to investigate the sustainability of off-treatment responses among Chinese HBeAg-positive CHB patients treated with PEG-IFN alfa-2b from a randomized trial.

**Methods:**

Eligible Chinese patients (*n* = 322) were followed up by one visit after a median of 6 years (LTFU) following their participation in a randomized trial evaluating the efficacy of three PEG-IFN alfa-2b dosing regimens (1.0 or 1.5 μg/kg/wk. 24 weeks or 1.5 μg/kg/wk. 48 weeks). Primary endpoints at the LTFU were sustained SR and CR (SR/CR at the end of original study [EOS] and at the LTFU). SR was defined as HBeAg loss and seroconversion to anti-HBe and CR as HBeAg loss and seroconversion to anti-HBe and HBV-DNA < 2000 IU/mL.

**Results:**

The proportions of patients achieving sustained SR among patients who had SR at EOS were high in three treatment groups (61.9, 65.5, 76.5%, respectively, *p* = 0.46); treatment with PEG-IFN alfa-2b 1.5 μg/kg/wk. 48 weeks had the highest proportion of a sustained CR among patients who had CR at EOS (75.0%, *p* = 0.05). A considerable number of patients achieved sustained SR (18.2–29.9%) and sustained CR (14.8–18.3%) after EOS despite no further NA treatment. At the LTFU, rates of SR and CR were less than 70.0 and 50.0%, respectively, among all enrolled patients regardless of additional nucleos(t)ide analogs before the LTFU.

**Conclusions:**

PEG IFN alfa-2b therapy had considerable off-treatment sustainability in Chinese HBeAg positive chronic hepatitis B patients with serological and complete responses.

## Background

Approximately 240 million people worldwide are affected by chronic hepatitis B (CHB) [1]. Complications occur in approximately 20 to 30% of CHB patients, resulting in a CHB-related mortality rate of 650,000/year globally. In China, 90 million people are affected, accounting for almost 7% of the total population. The annual hepatitis B virus (HBV)-related cancer mortality rate in China is 330,000/year [2, 3]. Because CHB is a significant risk factor for cirrhosis and hepatocellular carcinoma (HCC), intensive efforts have focused on the development of effective therapeutic strategies that aim for the clearance of HBV as measured by hepatitis B surface antigen (HBsAg), hepatitis B e antigen (HBeAg), viral DNA, normalization of hepatic function indicated by alanine transaminase (ALT), and slowing the progression of cirrhosis and inflammation in the liver [4]. Current therapeutic guidelines recommend the use of oral nucleosides/nucleotide analogues (NAs) and interferon-based therapy with interferon-alpha or pegylated interferon-alpha for patients with HBeAg-positive CHB [5, 6].

NAs effectively suppress viral replication and reduce disease-related morbidity and mortality. NAs require long-term treatment and are associated with the development of drug resistance and high relapse rates [7]. Interferon-based therapy has a finite treatment course with no associated drug resistance, although more frequent adverse events and the route of administration are of clinical concerns [8–10]. The major advantage of pegylated interferon (PEG-IFN) therapy is the induction of a robust off-treatment response along with increased virological response during the follow-up period, reflecting a post-treatment delayed immune response [11]. Randomized controlled trials (RCT) have demonstrated PEG-IFN treatment resulted in HBeAg seroconversion in approximately one-third of HBeAg-positive CHB patients, including those who were previously treated [12–14].

Studies investigating the long-term clinical responses to PEG-IFN in Europe, Asia, and the USA have reported sustained response rates ranging from 21 to 60%, depending on the treatment regimen, the definition of response, and the length of follow-up [1, 15–17]. Some CHB patients, nevertheless, had treatment relapse after a certain period of post-treatment observation, especially among those co-infected with HBV and HCV [14, 18, 19]. Thus, evaluating the sustainability of treatment response to PEG-IFN treatment in the long term is in need for patients with CHB.

In China, evidence of a long-term follow-up (LTFU) on PEG-IFN off-treatment response among HBeAg positive patients is very limited. Findings from an Asian multicenter RCT (P05170) favored PEG-IFN alfa-2b dosing scheme by 1.5 μg/kg/wk. for 48 weeks over other dosing schemes by 1.0 or 1.5 μg/kg/wk. for 24 weeks for HBeAg seroconversion among patients with CHB (with > 95% were Chinese) [20]. Therefore, this RCT provided us an opportunity to investigate the sustainability of complete response (CR) and serological response (SR) in Chinese CHB patients treated with the different PEG-IFN alfa-2b dosing schemes in a relatively longer off-treatment duration [20].

## Material and methods

### Study design and patients

This study was designed as a LTFU for the original multicenter, randomized, controlled study (P05170) [20] in which 657 interferon-naïve CHB patients were randomized to receive different PEG-IFN alfa-2b regimens (1.0 μg/kg/week for 24 weeks; 1.5 μg/kg/week for 24 or 48 weeks) followed by 24 weeks of post-treatment observation [20]. The LTFU was conducted at 21 out of 25 P05170 sites in China. Enrollment began in May 2014 and ended in November 2015 due to no potential to recall any more subjects from P05170. MSD China sponsored the study and designed and analyzed the study in collaboration with leading investigators. This study was conducted in accordance with the guidelines of the Declaration of Helsinki and the principles of Good Clinical Practice (ICH-E6) and was approved by independent ethics committees of all study sites before the study enrollment commenced. Medical Ethics Committee of NanFang Hospital of Southern Medical University (NFEC-201402-Y2).

Patients were eligible to the LTFU if they were randomized and treated at the sites from P05170 in mainland China and willing to give consent. Patients were excluded if they were pregnant or nursing or had a history of any illness that, in the opinion of the investigator, might affect the completion of LTFU. Subjects qualified for the LTFU were contacted by telephone or physical visit upon the approval from the local ethics committees and were screened by the investigator at an outpatient hepatology clinic in the participating sites. All patients completed the LTFU by one onsite visit once included. A separate and specific signed informed consent was obtained from all patients prior to study screening for participation in the LTFU.

### Clinical and laboratory measurements

The clinical and laboratory measurements of P05170 were reported previously [20]. Briefly, assays for HBV-DNA, HBV genotyping and virology, and clinical assessment, including liver biopsies, were conducted at baseline, at the end of treatment, and at a 24-week post-treatment visit for all randomized patients.

During the LTFU, the investigator assessed clinical signs, symptoms, and complications of liver disease (i.e., hepatocellular carcinoma, ascites, variceal bleeding, encephalopathy, jaundice, or liver transplant), and the administration of (any other) antiviral therapy after the end of P05170. Prior and concomitant medications at the LTFU for the treatment of CHB and/or other active medical conditions were documented. Co-medications dated during the period of original study observation were cross-checked by data records from P05170 [20]. Any supporting documents, including previous test results, were provided to the investigator to assess the clinical significance of additional antivirals after the end of the original study.

Blood samples for virological biomarkers (HBV-DNA, HBsAg/Ab, and HBeAg/Ab) were collected at the LTFU and sent to a central laboratory for measurement (LabCorp, Beijing, China). HBV DNA quantification at LTFU was performed using Taqman-based polymerase chain reaction (PCR) assays (COBAS AmpliPrep/COBAS TaqMan48, Roche Molecular Systems Inc.) with a lower detection limit of 20 IU/mL. Quantification of HBsAg levels were measured with Elecsys HBsAg II quantitative electrochemiluminescence immunoassay kits (Roche Diagnostics, Indianapolis, IN) using Modular Analytics E170 (Roche Diagnostics, Switzerland) with a range of 5–52,000 IU/mL for up to 400-fold dilutions of samples. HBeAg levels were quantified using the Elecsys HBeAg II quantitative assay using Modular Analytics E170 with a lower quantification limit of 5 PEIU/mL. Hematology and chemistry were measured at the participating sites using local laboratory standards.

### Clinical endpoints

The study endpoints were sustained complete response (CR) and sustained serological response (SR) at the LTFU. Sustained serological response was defined as HBeAg loss and seroconversion to anti-HBe at the end of the P05170 study and at the LTFU. Sustained complete response was defined as HBeAg loss and seroconversion to anti-HBe as well as HBV-DNA < 2000 IU/mL at the end of P05170 study and at the LTFU.

### Statistical analysis

#### Sample size

The rate of subject recall from P05170 determined the sample size for the LTFU rather than any sample size consideration based on inference statistic and power calculation. It was anticipated that 60% of subjects from the full analysis set (FAS) in P05170 would be included in the LTFU, representing a total sample size of 395 subjects. Study enrollment stopped when 322 subjects were screened and included in the LTFU, as the study team concluded that there was no potential to find any more subjects from the original study, albeit all practiced efforts.

### Analysis of endpoints

A point estimate (proportion) for the study endpoints was presented by different treatment schemes groups. Off-treatment sustainability was calculated as the number of patients who had sustained SR or CR at the LTFU divided by the number of patients who had SR or CR at EOS (of P05170). The duration of follow-up after the end of the P05170 study was calculated as the interval between the date of the last visit in P05170 and the date of LTFU visit. Missing data for study endpoints were not imputed by any means. Subjects with missing data for study endpoint assessment were considered non-responders for study endpoints. Subjects who were clinically re-treated with any antiviral therapy before and at the time of the LTFU were deemed non-responders for study endpoints. Univariate Cox regression analysis was used to identify factors associated with a sustained SR or CR in LTFU. Hazard ratio (HR) and 95% confidence interval (CI) were calculated to quantify the strength of associations. Variables with a *p* < 0.25 by Wald test for regression coefficient were used to perform multivariate Cox regression analysis using a forward selection procedure.

The subject’s clinical and laboratory characteristics are summarized and presented by different time points in P05170 and at the LTFU. Continuous variables are presented as the mean (standard deviation, SD); categorical variables are expressed as count and frequency (%). Group differences were compared using analysis of variance (ANOVA) or χ^2^ test whenever appropriate. All statistical analyses were performed using NCSS 10 software (NCSS, LLC, Kaysville, UT). All statistical tests were two-sided and performed at the 0.05 level of significance unless otherwise specified.

## Results

### Study population

**Fig. 1:**
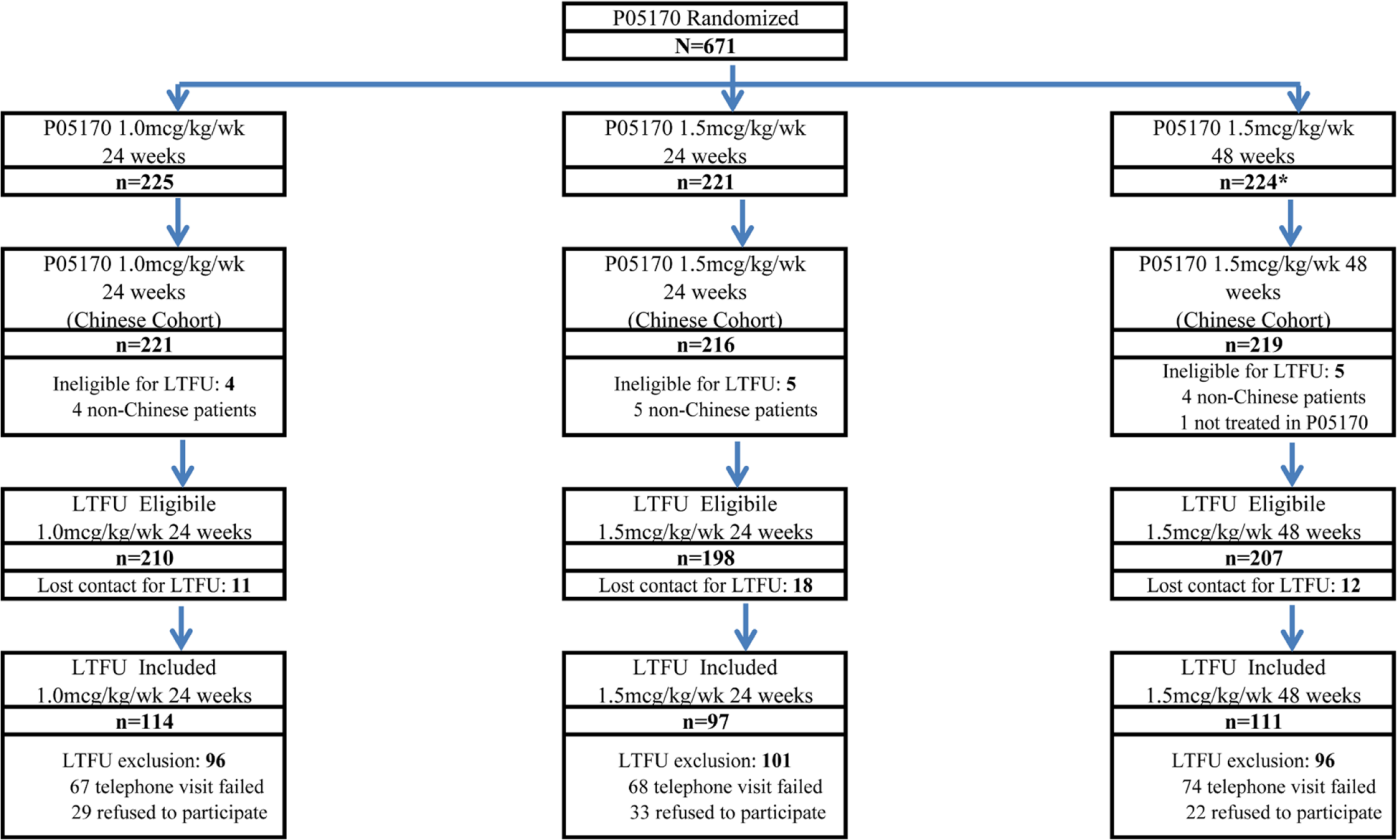
Patient diagram for long-term follow-up study following the P05170 multicenter randomized trial. In the long-term follow-up (LTFU), 93.6% (615/657) of subjects from PEG-IFN alpha-2b therapy were contacted by a telephone interview. Of the 615 subjects contacted, 34.0% (209/615) of subjects failed to connect, 13.7% (84/615) declined the LTFU. Hence, a total of 322 subjects had a site visit after the telephone interview, and completed the study. The overall LTFU rate for the study was 49.0% (322/657). No subject (0/322) withdrew during the LTFU study. *One patient received no study medication after randomization. “Lost contact” indicates the investigators did not have any contact information on the subject from P05170, and “Telephone visit failed” indicates patients who were called but no one answered the phone or the number was incorrect

A total of 615 subjects from P05170 who resided in mainland China were qualified for telephone or physical recall to participate in the LTFU by 1 onsite visit. Of these, 209 (34.0%) failed to connect and 84 (13.7%) declined the LTFU via phone. A total of 322 patients (1.0 μg/kg/wk. 24 weeks: 114, 1.5 μg/kg/wk. 24 weeks: 97, 1.5 μg/kg/wk. 48 weeks: 111) were finally included in the LTFU onsite visit, representing a recall rate of 49.0% (Fig. 1).

### Demographic and clinical characteristics

Demographic and clinical characteristics for all 322 subjects at P05170 baseline, the end of treatment (EOT), and the end of study (EOS) through their participation in the LTFU are presented in Table 1. At baseline, the mean age of the patient population was 28.9 years; most patients were male (78.3%), and 44.4 and 54.0% of patients had HBV genotype B or C, respectively. Overall, there are no significant differences observed for characteristics and endpoints among the three treatment groups at different study time points including the LTFU. At the EOS of P05170, a significantly higher proportion of patients who had ALT normalization was seen in the treatment group by 1.5 μg/kg/wk. for 48 weeks (44.1%, *p* = 0.01 for comparisons); patients treated by 1.5 μg/kg/wk. for 48 weeks had a markedly higher rate of HBV-DNA < 2000 IU/mL (26.1%, *p* = 0.03 for comparisons). The proportions of patients who had HBe seroconversion and ALT normalization or HBe seroconversion and HBV-DNA < 2000 IU/mL are generally low and do not differ statistically among three treatment groups (*p* = 0.05, *p* = 0.06, respectively) at the EOS.

**Table 1:** Clinical and Laboratory Characteristics for Subjects Enrolled in both the Original and LTFU Studies

	PEG-IFN alfa-2b 1.0 μg/kg/wk 24 weeks	PEG-IFN alfa-2b 1.5 μg /kg/wk 24 weeks	PEG-IFN alfa-2b 1.5 μg /kg/wk 48 weeks	Total	*p*
	*n* = 114	*n* = 97	*n* = 111	*n* = 322	
**Baseline**					
Age (Years)	28.5 (7.5)	28.9 (6.8)	29.3 (8.1)	28.9 (7.5)	0.77
Sex (Male)	89 (78.1)	77 (79.4)	86 (77.5)	252 (78.3)	0.94
BMI	22.4 (2.8)	22.2 (3.2)	22.7 (3.5)	22.4 (3.2)	0.48
HBV Genotype					0.83
B	48 (42.1)	43 (44.3)	52 (46.8)	143 (44.4)	
C	64 (56.1)	53 (54.6)	57 (51.4)	174 (54.0)	
Other	2 (1.8)	1 (1.0)	2 (1.9)	5 (1.5)	
Total HAI Score	8.0 (3.1)	8.0 (2.9)	8.0 (3.1)	8.0 (3.0)	0.99
ALT (*ULN)	4.1 (1.9)	4.4 (2.5)	4.0 (2.0)	4.1 (2.1)	0.41
ALT≥3*ULN	69 (60.5)	57 (58.8)	70 (63.1)	196 (60.9)	0.67
HBV DNA (LogIU/mL)	7.7 (0.9)	7.8 (0.7)	7.8 (0.8)	7.8 (0.8)	0.69
HBsAg (Log10 IU/mL)	4.2 (0.7)	4.2 (0.6)	4.3 (0.6)	4.2 (0.6)	0.31
HBeAg (Log10 IU/mL)	2.5 (0.7)	2.4 (0.7)	2.6 (0.7)	2.5 (0.7)	0.07
**P05170 EOT**					
ALT Normalization	33 (28.9)	36 (37.1)	49 (44.1)	118 (36.6)	0.06
HBeAg negativity	14 (12.3)	17 (17.5)	19 (17.1)	50 (15.5)	0.49
HBe seroconversion	14 (12.3)	17 (17.5)	19 (17.1)	50 (15.5)	0.49
HBV-DNA < 2000	13 (11.4)	14 (14.4)	18 (16.2)	45 (14.0)	0.57
HBsAg any decrease	87 (76.3)	66 (68.0)	86 (77.5)	239 (74.2)	0.25
**P05170 EOS**					
ALT Normalization	29 (25.4)	35 (36.1)	49 (44.1)	113 (35.1)	0.01
HBeAg negativity	21 (18.4)	21 (21.6)	34 (30.6)	76 (23.6)	0.08
HBe seroconversion	21 (18.4)	20 (20.6)	34 (30.6)	75 (23.3)	0.07
HBV-DNA < 2000	17 (14.9)	13 (13.4)	29 (26.1)	59 (18.3)	0.03
HBsAg any decrease	71 (62.3)	58 (59.8)	80 (72.1)	209 (64.9)	0.14
HBe seroconversion+ALT normalization	13 (11.4)	15 (15.5)	26 (23.4)	54 (16.8)	0.05
HBe seroconversion+DNA < 2000	10 (8.8)	9 (9.3)	20 (18.0)	39 (12.1)	0.06
**LTFU^**					
Duration of Follow-up	5.7 (0.4)	5.7 (0.4)	5.2 (0.3)	5.6 (0.4)	< 0.01
ALT (*ULN)	0.9 (0.8)	0.8 (0.6)	0.8 (0.8)	0.8 (0.7)	0.21
ALT Normalization	49 (43.0)	41 (42.3)	41 (36.9)	131 (40.7)	0.61
HBe Negativity	45 (39.5)	43 (44.3)	45 (40.5)	133 (41.3)	0.76
HBe Seroconversion	39 (34.2)	36 (37.1)	39 (35.1)	114 (35.4)	0.91
Additional Antivirals†	49 (43.0)	41 (42.3)	53 (47.7)	143 (44.4)	0.68

Data are presented as the mean (SD) or *n* (%); All analyses on endpoints and laboratory measurements in the original and LTFU studies were based on 322 subjects who were included in the LTFU; Including Genotype A, D or unidentified

^ Response for endpoints at LTFU was assessed as treatment response without any additional antivirals

Δ Calculated as time interval between the date of long-term follow-up visit and the date of P05170 off-treatment follow-up visit as expressed by the median (SD)

†Assessed as clinically significant antiviral treatment affecting clinical response assessment at LTFU before or during long-term follow-up visit; patients who were erroneously given nucleos(t)ide analogs (NA) during P05170 or initiated NAs at LTFU were excluded

*EOT* End of Treatment, *EOS* End of Study, *LTFU* Long-Term Follow-Up

All subjects were followed for up to 6 years (median, 5.6; SD, 0.4) at the LTFU from P05170. At LTFU, the overall percentages of patients who had ALT normalization, HBe negativity or HBe seroconversion were 40.7, 41.3, and 35.4%, respectively with no observed statistical significance across treatment groups (all *p* values> 0.60). Notably. The proportion of patients who achieved HBe seroconversion increased overtime from the P05270 EOT (12.3%), EOS (18.4%) to the LTFU (34.2%) in the group treated by 1.0 μg/kg/wk. for 24 weeks. A similar trend in HBe seroconversion was observed in the group treated by 1.5 μg/kg/wk. for 24 weeks (EOT: 17.5%, EOS: 20.6%, LTFU: 37.1%) but not in the group treated by 1.5 μg/kg/wk. for 48 weeks (EOT: 17.1%, EOS: 30.6%, LTFU: 35.1%) (Table 1). The proportions of patients treated by additional antivirals appeared comparable across treatment groups (*p* = 0.68).

### Sustained serological response and sustained complete response

Results of sustained SR and sustained CR at the LTFU are exhibited in the Table 2. Sustainability of off-treatment SR from EOS to the LTFU appeared generally high among 3 PEG-IFN alfa-2b dosing groups. Treatment with PEG-IFN alfa-2b 1.5 μg/kg/wk. for 48 weeks had a numerically higher sustainability in SR (76.5%, *p* = 0.46) compared with the other two treatment groups. Sustainability of off-treatment CR from EOS to the LTFU was markedly lower in the groups treated by PEG-IFN alfa-2b 1.0 or 1.5 μg/kg/wk. for 24 weeks (40.0, 33.3%, respectively) as compared to the group treated by PEG-IFN alfa-2b 1.5 μg/kg/wk. for 48 weeks (75.0%).

**Table 2:** Clinical Endpoints (SR/CR) in the Long-Term Follow-Up Study

	PEG-IFN alfa-2b 1.0 μg /kg/wk. 24 weeks	PEG-IFN alfa-2b 1.5 μg /kg/wk. 24 weeks	PEG-IFN alfa-2b 1.5 μg /kg/wk. 48 weeks
**P05170**	**225**	**221**	**224**
SR at EOS	38 (16.9)	36 (16.3)	67 (29.9)
CR at EOS	18 (8.0)	23 (10.4)	45 (20.1)
**Eligible for LTFU**	**210**	**198**	**207**
**Included for LTFU**	114 (54.3)	97 (49.0)	111 (53.6)
SR at EOS	21 (18.4)	20 (20.6)	34 (30.6)
CR at EOS	10 (8.8)	9 (9.3)	20 (18.0)
No additional NAs	65 (57.0)	56 (57.7)	58 (52.3)
Treated with NAs	49 (43.0)	41 (42.3)	53 (47.8)
Sustained response from EOS*			
SR at LTFU#	13 (61.9)	13 (65.5)	26 (76.5)
SR at LTFU with NA	17 (81.0)	17 (85.0)	30 (88.2)
NA for SR relapse	8 (38.1)	7 (35.0)	8 (23.5)
CR at LTFU	4 (40.0)	3 (33.3)	15 (75.0)
CR at LTFU with NA	5 (50.0)	4 (44.4)	16 (80.0)
NA for CR relapse	6 (60.0)	6 (66.7)	5 (25.0)
**EOS non-SR patients§**	**93**	**77**	**77**
New SR at LTFU	27 (29.0)	23 (29.9)	14 (18.2)
New SR at LTFU with NA	45 (48.4)	38 (49.4)	32 (41.6)
**EOS non-CR patients§**	**104**	**88**	**91**
New CR at LTFU	19 (18.3)	13 (14.8)	14 (15.4)
New CR at LTFU with NA	35 (33.7)	30 (28.8)	34 (32.7)

Overall data are presented as *n* (%). Chi-square test was used for statistical comparisons of SR and CR at the LTFU respectively

*Sustained response from EOS = patients with a response at EOS and response at LTFU/patients with response at EOS; *p* = NS for comparisons on SR or CR relapse

#*p* = 0.46 for SR at LTFU; Sustained SR at LTFU among all patients included in the LTFU in 3 groups: 11.4, 13.4, 23.4%, *p* = 0.03

*p* = 0.05 for CR at LTFU; Sustained CR at LTFU among all patients included in the LTFU in 3 groups: 3.5, 3.1, 13.5%, *p* < 0.01

§Patients who failed SR or CR at the EOS; *p* = NS for comparisons on new SR or new CR

*SR* HBeAg loss and seroconversion to anti-HBe, *CR* HBe seroconversion and HBV-DNA less than 2000 IU/mL, *relapse* patients who had SR/CR non-response at LTFU or were treated with additional antivirals after EOS, *EOS* end of (P05170) study, *LTFU* long-term follow-up; *NA* nucleos(t)ide analogs, *SR* serological response; *CR* complete response

Sustainability of SR or CR from EOS to the LTFU was numerically higher across three treatment groups when patients who had additional NAs to achieve SR or CR were included at the LTFU. On the other hand, a small fraction of patients who failed SR or CR at EOS became SR (< 30%) or CR (< 20%) at the LTFU was observed among three treatment groups with no additional antiviral therapies between EOS and the LTFU. Rates of new SR or CR were relatively higher when additional NAs were administered among all three treatment groups.

### Clinical responses at LTFU grouped by additional NAs

Different clinical endpoints at the LTFU, regardless of previous outcomes, were analyzed in patients included in the LTFU by the stratification according to treatment of additional NAs (Table 3). Higher rates of ALT normalization at the LTFU were seen among patients who had additional NAs in three treatment groups. Patients treated with additional NAs had a higher rate of HBsAg decline in the group treated with PEG-IFN alfa-2b 1.0 μg/kg/wk. 24 weeks (61.2 vs. 49.2%). Rates of SR at the LTFU were higher in the patients who were free of additional NAs in the three treatment groups and was the highest in the group treated by PEG-IFN alfa-2b 1.5 μg/kg/wk. 48 weeks (69.0% vs. 41.5%). The highest rate of CR was seen in the group treated by PEG-IFN alfa-2b 1.5 μg/kg/wk. 48 weeks without any additional NAs (50.0% vs. with NAs 39.6%). It is noted that the overall rate of SR and CR at the LTFU was < 70, 50%, respectively, among the three treatment groups with or without additional NAs before the LTFU visit.

**Table 3:** Clinical Endpoints in the Long-term Follow-up Study as Grouped by Additional NA Treatment

	PEG-IFN alfa-2b 1.0mcg/kg/wk 24 weeks		PEG-IFN alfa-2b 1.5mcg/kg/wk 24 weeks		PEG-IFN alfa-2b 1.5mcg/kg/wk 48 weeks		Total	
	Without NAs *n* = 65	Additional NAs *n* = 49	Without NAs *n* = 56	Additional NAs *n* = 41	Without NAs *n* = 58	Additional NAs *n* = 53	Without NAs *n* = 179	Additional NAs *n* = 143
HBe Negativity	46 (70.8)	30 (61.2)	43 (76.8)	23 (56.1)	46 (79.3)	31 (58.5)	135 (75.4)	84 (58.7)
ALT Normalization	48 (73.8)	38 (77.6)	40 (71.4)	39 (95.1)	41 (70.7)	45 (84.9)	129 (72.1)	122 (85.3)
HBe(−) + ALT Normalization	36 (55.4)	26 (53.1)	35 (62.5)	22 (53.7)	38 (65.5)	29 (54.7)	109 (60.9)	77 (53.8)
SR	40 (61.5)	22 (44.9)	36 (64.3)	19 (46.3)	40 (69.0)	22 (41.5)	116 (64.8)	63 (44.1)
CR	23 (35.4)	17 (34.7)	16 (28.6)	18 (43.9)	29 (50.0)	21 (39.6)	68 (38.0)	56 (39.2)
HBsAg any decrease ^	32 (49.2)	30 (61.2)	23 (41.1)	18 (43.9)	34 (58.6)	31 (58.5)	89 (49.7)	79 (55.2)
Decompensated Liver Event*	0 (0.0)	0 (0.0)	0 (0.0)	1 (2.4)	0 (0.0)	0 (0.0)	0 (0.0)	1 (0.7)

Serological response (SR) was defined as Hbe seroconversion (HBeAg negativity and HBeAb positivity)

Complete clinical response (CR) was defined as Hbe seroconversion (HBeAg negativity and HBeAb positivity) and HBV-DNA less than 2000 IU/mL

^Defined as any decrease of HBsAg quantification levels at LTFU from that in the end of P05170 study observation;

*1 patient had liver biopsy and clinically confirmed liver cirrhosis

*LTFU* long-term follow-up, *NA* nucleos(t)ide analogs, *SR* serological response, *CR* complete response

### Univariate and multivariate analysis

Univariate and multivariate Cox regression analysis was used to identify factors associated with SR and CR (Table 4). Multivariate Cox regression analysis showed that patients with HBV genotype B were significantly less likely to have a sustained SR compared with those harboring other HBV genotypes (HR, 0.42; 95% CI: 0.22–0.81; *p* = 0.01). Patients treated with 1.5 μg/kg/wk. for 48 weeks had a 5-fold higher likelihood of achieving a sustained SR compared with those treated with a 24-week PEG-IFN dosing scheme (HR, 5.00; 95% CI: 2.50, 10.00; *p* < 0.01). Patients treated with a regimen of 1.5 μg/kg/wk. for 48 weeks had a 7.7-fold higher likelihood of achieving sustained CR compared with those who received the other two dosing schemes (95% CI: 2.00, 25.00; *p* < 0.01).

**Table 4:** Univariate and Multivariate Cox Regression Analyses Exploring Associations between A Sustained SR or Sustained CR and Baseline/On-Treatment/Off-Treatment Characteristics

	Sustained Serological Response				Sustained Complete Clinical Response			
	Univariate		Multivariate		Univariate		Multivariate	
	HR (95% CI)	*p*	HR (95% CI)	*p*	HR (95% CI)	*p*	HR (95% CI)	*p*
Baseline								
Age	1.01 (0.99, 1.02)	0.44			1.01 (0.94, 1.07)	0.82		
Gender (Male)	1.24 (0.60, 2.55)	0.57			1.40 (0.84, 2.34)	0.20		
BMI	1.10 (0.97, 1.25)	0.13			0.97 (0.82, 1.15)	0.73		
HBV Genotype (B)	0.63 (0.36, 1.13)	0.12	0.42 (0.22, 0.81)	0.01	0.50 (0.18, 1.36)	0.17		
ALT< 3*ULN	1.15 (0.58, 2.26)	0.70			0.99 (0.35, 2.78)	0.99		
HBsAg (Log10 IU/mL)	0.97 (0.63, 1.52)	0.91			1.00 (0.45, 2.21)	0.99		
HBeAg (Log10 IU/mL)	1.29 (0.84, 1.98)	0.24			1.36 (0.68, 2.74)	0.39		
HBV DNA (Log10 IU/mL)	0.96 (0.69, 1.31)	0.78			0.83 (0.44, 1.56)	0.56		
PEG-IFN alfa-2b (48-week scheme)	3.69 (1.96, 6.92)	< 0.01	5.00 (2.50, 10.00)	< 0.01	7.69 (2.00, 25.00)	< 0.01	7.07 (1.87, 26.79)	< 0.01

All baseline and selected on treatment characteristics were analyzed (*N* = 322) by univariate analysis and variables with a *p*-value< 0.25 entered into multivariate model where variables were eliminated by hierarchical forward with switching method until p values for all retained variables were less than 0.1; Wald test method was used to give p values for variable’s regression coefficient

Sustained serological response was defined as Hbe seroconversion at the end of P05170 and in the LTFU

^†^All patients who have a sustained response were free of any additional antivirals. No actual HR and 95%CI will be calculated

^‡^Not applicable: the duration of follow-up by year is deemed as having a probability distribution in survival function and was not incorporated into the Cox model as an independent variable

## Discussion

This long-term follow-up study was an extension of our previous study [20] and compared the sustainability of three PEG-IFN alfa-2b dosing schemes for the treatment of Chinese HBeAg-positive patients with CHB six years post-treatment. Findings from the study confirmed a good sustainability of PEG-IFN alfa-2b off-treatment response in the long term and the most efficacious was seen in the patient group treated with 1.5 μg/kg/wk. for 48 weeks. Other important findings suggested an increase in the rates of HBe seroconversion over time in patients receiving PEG-IFN alfa-2b 24-week dosing schemes and overall low-to-moderate rates of SR and CR in patients at the LTFU in all treatment groups with or without additional NA therapies.

The findings of the present study are consistent with earlier studies that investigated the sustainability of treatment response in which duration of treatment was found as an important factor associated with the long-term durability of PEG-IFN therapy in HBeAg-positive patients [8, 14, 15, 21–24]. The response rates in this study are comparable to those reported previously [8, 14, 15, 21–24]. Wong et al. (2010) found that the response (HBe seroconversion with HBV-DNA < 10,000 IU/mL) to PEG-IFN alpha-2b was sustained for five years in HBeAg-positive CHB patients; 82 and 57% had sustained HBeAg seroconversion and virologic response, respectively, at the end of follow-up [21]. Okanoue et al. (2016) showed that patients with CHB infection (*N* = 137) sustained a good response (normal ATL levels and low-level of hepatitis B virus) five years post-PEG-IFN alpha-2a treatment, and a 48-week administration of PEG IFN-α-2a showed a better response rate (26.4%) than a 24-week administration (18.0%) [22]. However, the study revealed that > 60% of patients received additional antiviral therapy due to viral reactivation [22]. In a small Japanese study, Masaki et al. (2015) also found that a 48-week regimen of PEG-IFN alfa-2a had significantly higher rates of HBeAg clearance, ALT normalization, and HBV-DNA loss at the end of five years [15].

Considering additional antivirals before the LTFU, we evaluated the relapse rates in patients who achieved SR or CR at the end of the original study and the clinical endpoints in patient subgroups with or without additional NAs at the LTFU time point. Patients treated with the 48-week PEG IFN alfa-2b dosing scheme had the lowest need for additional NA therapy. However, additional NA treatment after initial PEG-IFN dosing showed better response rates in normalizing ALT and slightly higher response rates in reducing HBsAg load for patients receiving 24-week PEG-IFN dosing schemes. It should be cautious in the clinical practice that overall rates of SR and CR were generally not satisfactory for CHB patients at the LTFU. On the other hand, we found SR/CR response rates between 18 and 30% at the LTFU for patients who had failed such responses at the end of original study but were free of additional NAs, which were similar to results reported from a previous study [25]. In particular, patients who received a 24-week dosing scheme had an increase in HBe seroconversion from the end of previous study treatment through the LTFU. These findings further supported a longer-term immunomodulatory effect with PEG-IFN treatment in at least in a small fraction of HBeAg-positive CHB patients.

A number of studies have investigated baseline and early on-treatment predictive factors associated with the durability of response to PEG-IFN therapy. Early HBeAg loss after PEG-IFN alfa-2b therapy was associated with viral DNA suppression and HBsAg loss at a 3-year LFTU visit [26]. Sonneveld et al. (2010) found that 97% of HBeAg-positive CHB patients treated with PEG-IFN who showed no decrease in serum HBsAg levels at 12 weeks of treatment showed a non-response at three years [27]. Additionally, Sonneveld et al. (2012) demonstrated that PEG-IFN alfa-2b-treated CHB patients who had a complete response (HBeAg loss along with HBV DNA < 10,000 copies/mL) showed a sustained HBsAg decline after three years [28]. Ma et al. (2010) showed that on-treatment serum HBsAg and HBeAg levels were significant predictors of sustained response to PEG-IFN alfa-2b treatment for CHB [24] which was supported by a study from Wang et al. (2016) [29].

Virological load has also been shown to be a significant predictor for sustained treatment responses [21]. Okanoue et al. found that low HBV-DNA levels at the end of treatment were independently associated with favorable five-year response rates [22]. A long-term follow-up study of 21 Taiwanese HBeAg-positive patients with CHB at 6.5–12.5 years of IFN-alpha post-treatment found that pre-treatment with HBV DNA load (< 2 × 10^8^ copies/mL) predicted sustained cumulative virological response [30]. Other factors including younger age and baseline ALT levels were explored to have an association with a sustained virological response [31].

We created Cox regression models to explore baseline and on-treatment predictors, including demographics and CHB biomarkers for sustained SR and CR. Patients carrying genotype B HBV were significantly less likely to have a sustained SR compared to those carrying other genotypes (genotype C: 54.0%). In addition, the dosing scheme by 1.5 μg/kg/wk. for 48 weeks was the only associated factor for sustained CR at the LTFU. Our results indicate that genotype B is associated with a reduced chance of sustained SR compared with other genotypes, which differs from the findings of previous studies. Zhao et al. (2007) found that genotype B and younger patient age (< 25 years) were independently associated with sustained CR and a better response to low-dose therapy among Chinese CHB-infected patients who were HBeAg positive (*N* = 230) and had received PEG-IFN alpha-2b therapy for 24 weeks [32]. In addition, Buster et al. (2008) found that the durable loss of HBeAg three years after PEG-IFN treatment was greater in patients carrying genotypes A and B than in those carrying genotypes C and D [26]. A pooled analysis of two large trials of HBeAg-positive patients treated with PEG-IFN found that higher levels of ALT and lower levels of HBV-DNA were predictive of sustained response for patients infected with genotypes A, B, and C [33] and genotype D had the lowest chance of sustained response, irrespective of HBV-DNA or ALT levels. In addition, Chen et al. (2011) found that genotype B was associated with better immediate, late, and sustained response at 24 weeks of follow-up in HBeAg-positive patients treated with PEG-IFN alpha-2a for six months [34]. The reasons for the differences between our findings and those of previous studies are not clear but may reflect the fact that most of our patients were infected with HBV of genotype B or C (98.4%); hence, our results primarily reflect differences between these two genotypes.

To the best of our knowledge, the LTFU is the first study evaluating long-term clinical data in Chinese CHB patients treated with PEG-IFN alfa-2b who were followed through a 6-year (312-week) period. Our study patients were selected from those who had completed our previously described P05170 study [20]. Sustained serological and complete responses were pre-defined and assessed throughout the RCT (P05170) until the LTFU. The present study was able to capture clinical information on additional antiviral use during the follow-up period. Patients were deemed non-responders if additional NA therapy was initiated after the completion of the original study or had any missing data regarding clinical response assessment.

This LTFU study has certain limitations. First, fewer than half of patients from P05170 were recalled and included in the LTFU. Patients with uncontrolled HBV infections may have sought additional medical treatment at other facilities. Moreover, those who had good long-term responses may have chosen to ignore this follow-up contact. Therefore, the sustainability and long-term clinical outcomes may be over- or underestimated. The LTFU was designed as one follow-up visit rather than as a continuous follow-up protocol by specific time-intervals. We did not have the opportunity to assess the exact time point for relapse in patients who achieved previous favorable responses or patients who had favorable responses despite their previously failed clinical outcomes. Lastly, due to lower-than-anticipated recall rates for patients treated by different PEG IFN dosing schemes, liver-related events were not sufficiently assessed and thus, liver related complications may be underreported.

## Conclusion

In conclusion, PEG IFN alfa-2b therapy had considerable off-treatment sustainability in Chinese HBeAg positive chronic hepatitis B patients with serological and complete responses.

## Abbreviations

ALT: Alanine transaminase
CHB: Chronic hepatitis B
CR: Complete response
HBV: Hepatitis B virus
HCC: Hepatocellular carcinoma
LTFU: Long-term follow-up visit
PCR: Polymerase chain reaction
SR: Serological response
